# Isolation and characterization of equine influenza virus (H3N8) from an equine influenza outbreak in Malaysia in 2015

**DOI:** 10.1111/tbed.13218

**Published:** 2019-05-22

**Authors:** Xinyu Toh, Moi Lien Soh, Mee Keun Ng, Shew Choo Yap, Nurshilla Harith, Charlene Judith Fernandez, Taoqi Huangfu

**Affiliations:** ^1^ Centre for Animal and Veterinary Sciences, Professional and Scientific Services, Animal and Veterinary Service National Parks Board Singapore City Singapore

**Keywords:** amino acid alignment, equine influenza, H3N8, phylogenetic analysis

## Abstract

Equine influenza is a major cause of respiratory infections in horses and can spread rapidly despite the availability of commercial vaccines. In this study, we carried out molecular characterization of Equine Influenza Virus (EIV) isolated from the Malaysian outbreak in 2015 by sequencing of the HA and NA gene segments using Sanger sequencing. The nucleotide and amino acid sequences of HA and NA were compared with representative Florida clade 1 and clade 2 strains using phylogenetic analysis. The Florida clade 1 viruses identified in this outbreak revealed numerous amino acid substitutions in the HA protein as compared to the current OIE vaccine strain recommendations and representative strains of circulating Florida sub‐lineage clade 1 and clade 2. Differences in HA included amino acids located within antigenic sites which could lead to reduced immune recognition of the outbreak strain and alter the effectiveness of vaccination against the outbreak strain. Detailed surveillance and genetic information sharing could allow genetic drift of equine influenza viruses to be monitored more effectively on a global basis and aid in refinement of vaccine strain selection for EIV.

## 1 INTRODUCTION

Equine influenza virus (EIV), an influenza A virus belonging to the *Orthomyxoviridae* family, is a major cause of respiratory diseases in horses and can spread rapidly between naïve animals. Influenza A viruses are subtyped according to their surface glycoprotein haemagglutinin (HA) and neuramindase (NA). The HA mediates virus entry into the host cell by binding to the sialic acid receptors and mediating fusion of viral and host membranes (Dimitrov, 2004). On the other hand, the NA is involved in cleavage of sialic acid to aid virus release from the infected cells (Shtyrya, Mochalova, & Bovin, 2009).

There are two major subtypes, H7N7 and H3N8, which have been isolated from horses (Scholtens, Steele, Dowdle, Yarbrough, & Robinson, 1964; Sovinona, Tumova, Pouska, & Nemec, 1958; Waddell, Teigland, & Sigel, 1963). However, the equine H7N7 viruses are thought to be extinct as no H7N7 viruses have been isolated since 1979 (Webster, 1993). The first EIV H3N8 virus was isolated during a widespread outbreak in Miami in 1963 (Scholtens et al., 1964; Waddell et al., 1963) and H3N8 viruses have continued to circulate till today (Favaro et al., 2018; Rash et al., 2017; Woodward et al., 2014). In the late 1980s, EIV diverged into two antigenically distinct lineages (Daly et al., 1996; Worobey, Han, & Rambaut, 2014), American and Eurasian, and since then, the American lineage has further diverged into the Kentucky, South American and Florida sub‐lineage clades 1 and 2 (Bryant et al., 2009; Murcia, Wood, & Holmes, 2011). Antigenic and genetic information suggest that evolution of EIV is mainly based on the mutations found on the HA surface glycoprotein. While the NA gene sequences of clade 1 and clade 2 viruses were clearly distinguishable, the majority of published sequences are for HA, making it difficult to study evolution of the NA gene segment.

Between 2006 and 2009, Florida sub‐lineage clade 2 viruses were predominantly isolated from the equine influenza outbreaks in Europe while Florida sub‐lineage clade 1 viruses circulated in North America (Bryant et al., 2009). In recent years, both clades of EIV have caused massive outbreaks of equine influenza in various geographic locations. For example, clade 1 viruses were responsible for the outbreaks in Japan and Australia in 2007 (Cowled, Ward, Hamilton, & Garner, 2009; Yamanaka, Niwa, Tsujimura, Kondo, & Matsumura, 2008) whereas clade 2 viruses were involved in the Mongolia outbreak in 2008 and the India outbreak in 2009 (Virmani et al., 2010; Yondon et al., 2013). Furthermore, in the 2014 equine influenza outbreaks, clade 1 viruses were detected in the USA (OIE, 2015; Sreenivasan et al., 2018) while clade 2 viruses were detected in France, Germany, Ireland, Sweden and the UK (Fougerolle et al., 2017; Gildea et al., 2018; Rash et al., 2017).

Vaccination plays an important role in the prophylactic treatment against equine influenza. Vaccines offer protection by the induction of antibodies to viral surface glycoproteins, in particular HA (Dimitrov, 2004). Similar to other influenza viruses, EIV may undergo antigenic drift to evade antibody responses. Therefore, the effectiveness of vaccines is dependent on the antigenic differences between the vaccine strain and the outbreak strain (Laver et al., 1979; Park et al., 2009). As such, vaccine strains for equine influenza need to be up to date to offer optimal protection. With this in mind, a comprehensive surveillance programme and active collaborative efforts between the World Organization for Animal Health (OIE) Expert Surveillance Panel (ESP) on Equine Influenza Vaccine Composition and external laboratories have contributed immeasurably to the epidemiological data required for facilitation of the selection of appropriate vaccine strains by OIE. Based on the current OIE recommendations, it is not necessary to include an H7N7 virus or an H3N8 virus of Eurasian lineage as these viruses have not been detected in most recent surveillance programmes. In lieu, the OIE recommends that international vaccines should include a representative of both Florida clade 1 (A/eq/South Africa/04/2003‐like or A/eq/Ohio/2003‐like) and clade 2 (A/eq/Richmond/1/2007‐like) (OIE, 2017).

In August–September 2015, 25 Thoroughbred horses transiting in Singapore from a racing club in Malaysia were found to have influenza‐like clinical signs. All 25 horses had been vaccinated against EIV, although full details of vaccination were unavailable. Nasopharyngeal swabs were obtained from the horses and submitted to the Animal Health Laboratory (AHL) of the Agri‐Food & Veterinary Authority (AVA) for disease diagnosis. The affected horses were kept in isolation and empirically treated pending outcome of laboratory diagnostics. Since Singapore is free from equine influenza, the import of horses from Malaysia was suspended.

In this study, virus isolated from the Malaysia outbreak in 2015 were genetically characterized by sequencing of the HA and NA gene segments. The Florida clade 1 viruses identified in this outbreak have further diverged as compared to the current OIE vaccine strain recommendations, pointing to a potential vaccine breakdown. Furthermore, sequence differences found between representative strains of two circulating clades of the Florida sub‐lineage were also highlighted. Information from this report could allow genetic drift of equine influenza viruses to be monitored more effectively on a global basis.

## 2 MATERIALS AND METHODS

Nasopharyngeal swabs were collected from 25 horses, exhibiting signs of acute respiratory illness. Each nasopharyngeal swab was collected using a swab with synthetic tip (e.g. Dacron) and a plastic shaft in 3 ml of Virus Transport Medium (Puritan UniTranz‐RT) and mixed vigorously using a vortex mixer to give the swab suspension before RNA extraction. RNA was isolated from 50 µl swab suspension using a MagMAX Total Nucleic Acid Isolation Kit (Thermo Fisher Scientific) as per the manufacturer's recommendation. The RNA was eluted in 50 µl elution buffer. Thereafter, a real‐time RT‐PCR (rRT‐PCR) assay (M+25 Forward Primer: 5′‐AGA TGA GTC TTC TAA CCG AGG TCG‐3′, M‐124 Reverse Primer: 5′‐TGC AAA AAC ATC TTC AAG TCT CTG‐3′, M+64 Probe: 5′‐[FAM]‐TCA GGC CCC CTC AAA GCC GA‐[TAMRA]‐3′) targeting the Influenza A matrix gene was used to detect the presence of EIV (Speckman et al., 2002). The rRT‐PCR assay was conducted in a 25‐µl reaction mixture containing 12.5 µl of 2 × RT‐PCR buffer (AgPath‐ID One‐Step RT‐PCR, Thermo Scientific), 1 µl of 25 × RT‐PCR enzyme mix, 1 µl (0.4 µmol) each of forward and reverse primers (10 µmol/L), 1 µl (0.24 µmol) of probe (6 µmol/L), 6 µl of nuclease free water and 2.5 µl of extracted RNA. The thermal profile consisted of an initial reverse transcription step at 45°C for 10 min, denaturation at 95°C for 10 min, followed by 40 cycles of denaturation at 95°C for 15 s and annealing at 60°C for 45 s. Ct values were used for classification of positive EIV‐RNA detections. Of the 25 samples received, the matrix gene was not detected in one of the samples.

The ESPLINE^TM^ Influenza A & B‐N Test Kit (Fujirebio) was also used as per the manufacturer's instructions as a rapid detection kit to confirm the presence of Influenza A antigens in the nasopharyngeal swab suspension.

Suspensions from nasopharyngeal swabs that were tested positive by the rRT‐PCR assay were cultivated in specific pathogen‐free (SPF) chicken‐embryonated eggs. Briefly, 200 μl of each swab suspension (neat) was inoculated into the allantoic cavity of five 9–11‐day‐old embryonated chicken eggs and incubated at 37°C. Three to five days later, eggs were chilled and the allantoic fluid was harvested and tested for EIV using the haemagglutination assay (Killian, 2014). If the allantoic fluid were positive in the haemagglutination assay, the virus was used for further characterization after EIV rRT‐PCR confirmation. In order to rule out the presence of EIV in the samples that were negative in the haemagglutination assay, the samples were subjected to a maximum of three passages in the eggs. Subsequently, PCR using EIV‐H3‐ and EIV‐H7‐specific primers were used to determine the HA subtype (Alves Beuttemmuller et al., 2016). The PCR products of the HA were subjected to Sanger sequencing (AITBiotech) for additional confirmation.

The full‐length sequences of H3 and N8 were mapped out using subtype‐specific primers elongated at the 5′ end by adding either M13 forward or M13 reverse primer sequences (Table 1) (Rash, Woodward, Bryant, McCauley, & Elton, 2014). Each segment was amplified in a 50‐µl PCR reaction consisting of 5 µl RNA using the Qiagen OneStep RT‐PCR kit. The one‐step RT‐PCR cycling protocol comprises of a reverse transcription step at 50°C for 30 min, an initial denaturation at 95°C for 15 min, followed by 35 cycles of denaturation at 94°C for 30 s, primer annealing at 50°C for 30 s, elongation at 72°C for 1 min and a final elongation at 72°C for 10 min. PCR reactions were analysed on a 1% agarose gel containing ethidium bromide stain according to manufacturer's instructions. PCR products were sequenced using Sanger sequencing.

**Table 1:** Primer sequences used to sequence the genome of H3N8 EIV (Rash et al., 2014)

Primer name	Primer sequence (5′−3′)
HA primers	
HA/AF	GCG TAA AAC GAC GGC CAG TAG CGA AAG CAG GGG ACG ATA TT
HA/AR	GCA ACA GCT ATG ACC ATG GAT TTG TTA GCC AAT TCA G
HA/BF	GCG TAA AAC GAC GGC CAG TCA GGT GTC ACT CAA AAC G
HA/BR	GCA ACA GCT ATG ACC ATG GGA TTT GCT TTT CTG GTA C
HA/CF	GCG TAA AAC GAC GGC CAG TGG TTA CAT ATG GAA AAT GCC
HA/CR	GCA ACA GCT ATG ACC ATG GAG CCA CCA GCA ATT CT
HA/DF	GCG TAA AAC GAC GGC CAG TGA AGG AAG AAT TCA GGA
HA/DR	GCA ACA GCT ATG ACC ATG GAG TAG AAA CAA GGG TGT TTT TAA C
NA primers	
NA/AF	GCG TAA AAC GAC GGC CAG TAG CAA AAG CAG GAG TTT
NA/AR	GCA ACA GCT ATG ACC ATG GCC CTA TTT TGA CAC TC
NA/BF	GCG TAA AAC GAC GGC CAG TCA CAC AGG GCT CAT TAC
NA/BR	GCA ACA GCT ATG ACC ATG CCG AAA CCT TTT ACA CCG
NA/CF	GCG TAA AAC GAC GGC CAG TCA CAG TTG GAT ATT TGT G
NA/CR	GCA ACA GCT ATG ACC ATG AGT AGA AAC AAG GAG TT

Nucleotide sequences were presented using SeqMan II version 5.03 (DNAstar Inc.). All sequences were deposited into GenBank (Accession numbers KT832068, KT832069 and KT867256). Nucleotide sequences were aligned to representative sequences for HA1 or NA sequences obtained from GenBank and Global Initiative on Sharing All Influenza Data (GISAID) (Shu & McCauley, 2017) database using MegAlign (DNAstar Inc.) (Table 2). Derived amino acid sequences were aligned against representative strains from each sub‐lineage of EIV, including Pre‐divergent, Eurasian, American and Florida clades 1 and 2. Phylogenetic analyses for the HA and NA nucleotide sequences and HA amino acid alignments were performed and created using MegAlign (DNAstar Inc.).

**Table 2:** Equine influenza viruses included in phylogenetic analysis. We gratefully acknowledge the originating and submitting laboratories of the sequences from GISAID's EpiFlu^TM^ Database on which this research is based

Strain name	Lineage	HA1^a^	HA protein^a^	NA^a^		Originating Laboratory	Submitting Laboratory
Fontainebleau/1/1979	Pre‐divergent	KJ643904.1	ACD85396.1	CY032407.1		–	–
Kentucky/2/1981	Pre‐divergent	CY028820.1	ABY81470.1	CY028822.1		–	–
Miami/1963	Pre‐divergent	M29257.1	AAA43164.1	CY028838.1		–	–
Newmarket/D64/1979	Pre‐divergent	D30677.1	BAA33938.1	–		–	–
Newmarket/1/1993	American	X85088.2	CAA59415.3	FJ375222.1		–	–
Kentucky/1/1992	American	CY030149.1	ACA24634.1	CY030151.1		–	–
Kentucky/1/1993	American	JN084405.1	AEI26214.1	–		–	–
Kentucky/1/1997	American	AF197249.1	AAF22353.1	–		–	–
Kentucky/1/1998	American	AF197241.1	AAF22345.1	–		–	–
Philippines/2/1997	American	JN084413.1	AEI26222.1	–		–	–
Newmarket/2/1993	Eurasian	X85089.2	CAA59416.3	FJ375223.1		–	–
Hong Kong/1/1992	Eurasian	L27597.1	AAA62470.1	–		–	–
Suffolk/1989	Eurasian	X68437.1	CAA48482.1	–		–	–
Sussex/1/1989	Eurasian	KJ643906.1	ACD97425.1	CY032319.1		–	–
Bridgend/1/2009	Florida clade 1	CY054289.1	ADM64575.1	–		–	–
Carlow/1/2009	Florida clade 1	JN222939.1	AEK98501.1	–		–	–
Cheshire/1/2009	Florida clade 1	GU045285.1	ACX48087.1	–		–	–
Cheshire/2/2009	Florida clade 1	GU045286.1	ACX48088.1	–		–	–
Concepcion/RO1C/2018	Florida clade 1	MH347131.1	AWW21162.1	MH346951.1		–	–
Curico/RO3B/2018	Florida clade 1	MH346560.1	AWW20326.1	MH346923.1		–	–
Donegal/1/2009	Florida clade 1	JN222938.1	AEK98500.1	–		–	–
Dorset/1/2009	Florida clade 1	CY054287.1	ADM64573.1	–		–	–
East Lothian/1/2018	Florida clade 1	EPI1212918		EPI1212919		Animal Health Trust, UK	Animal Health Trust, UK
Essex/1/2019	Florida clade 1	EPI1355455		EPI1355456		Animal Health Trust, UK	Animal Health Trust, UK
Georgia/121362−16/2016	Florida clade 1	MF173124.1	ASB31242.1	MF173198.1		–	–
Herefordshire/1/2009	Florida clade 1	GU045269.1	ACX48071.1	–		–	–
Ibaraki/1/2007	Florida clade 1	AB360549.2	BAG12477.2	LC369074.1		–	–
Kentucky/1/2009	Florida clade 1	CY054292.1	ADM64578.1	–		–	–
Kentucky/1/2011	Florida clade 1	KF026400.1	AGN51060.1	–		–	–
Kentucky/1/2012	Florida clade 1	KF026407.1	AGN51067.1	–		–	–
Lanarkshire/1/2009	Florida clade 1	CY054285.1	ADM64571.1	–		–	–
Limerick/1/2010	Florida clade 1	JN222940.1	AEK98502.1	–		–	–
Lincolnshire/1/2007	Florida clade 1	FJ195398.2	ACH95572.2	KF559342.1		–	–
Lincolnshire/1/2019	Florida clade 1	EPI1355457		EPI1355458		Rossdales Laboratories, UK	Animal Health Trust, UK
Monmouthshire/1/2009	Florida clade 1	CY054284.1	ADM64570.1	–		–	–
New York/1/2011	Florida clade 1	KF026399.1	AGN51059.1	–		–	–
New York/135857/2016	Florida clade 1	MF173262.1	ASB31259.1	MF173246.1		–	–
Nottinghamshire/1/2009	Florida clade 1	CY054286.1	ADM64572.1	–		–	–
Ohio/1/2003	Florida clade 1	DQ124192.1	ABA39846.1	DQ124168.1		–	–
Ohio/1/2008	Florida clade 1	GU045283.1	ACX48085.1	–		–	–
Ohio/1/2009	Florida clade 1	CY054290.1	ADM64576.1	–		–	–
Oklahoma/1/2008	Florida clade 1	GU045284.1	ACX48086.1	–		–	–
Pennsylvania/1/2007	Florida clade 1	FJ195406.1	ACH95580.1	–		–	–
Sao Paulo/IB19/2012	Florida clade 1	KC620391.1	AGG86717.1	–		–	–
Santiago/TT1A/2018	Florida clade 1	MH346818.1	AWW20712.1	MH346708.1		–	–
South Africa/4/2003	Florida clade 1	GU447312.1	ADB45165.1	–		–	–
Texas/1/2012	Florida clade 1	KF026410.1	AGN51070.1	–		–	–
Uruguay/2/2018	Florida clade 1	MH673717.1	AXF95018.1	–		–	–
Virginia/1/2008	Florida clade 1	CY054291.1	ADM64577.1	–		–	–
Yokohama/aq100/2017	Florida clade 1	LC269107.1	BAX51235.1	–		–	–
Aboyne/1/2008	Florida clade 2	GU045271.1	ACX48073.1	–		–	–
Aboyne/2/2008	Florida clade 2	GU045272.1	ACX48074.1	–		–	–
Donegal/1/2007	Florida clade 2	JN222934.1	AEK98496.1	–		–	–
Down/1/2008	Florida clade 2	JN222937.1	AEK98499.1	MG586817.1		–	–
Fife/1/2016	Florida clade 2	EPI957622		EPI957623		Animal Health Trust, UK	Animal Health Trust, UK
Gloucestershire/2/2016	Florida clade 2	EPI957813		EPI957814		Animal Health Trust, UK	Animal Health Trust, UK
Hawick/1/2008	Florida clade 2	GU045274.1	ACX48076.1	–		–	–
Hampshire/3/2016	Florida clade 2	EPI824114		EPI957607		Animal Health Trust, UK	Animal Health Trust, UK
Kent/1/2016	Florida clade 2	EPI957608		EPI957609		Animal Health Trust, UK	Animal Health Trust, UK
Kildare/1/2007	Florida clade 2	JN222936.1	AEK98498.1	–		–	–
Lancashire/1/2016	Florida clade 2	EPI957669		EPI957670	Animal Health Trust, UK		Animal Health Trust, UK
Lanarkshire/1/2008	Florida clade 2	GU045275.1	ACX48077.1	–		–	–
Lanarkshire/2/2008	Florida clade 2	GU045276.1	ACX48078.1	–		–	–
Lanarkshire/3/2008	Florida clade 2	GU045277.1	ACX48079.1	–		–	–
Lanarkshire/5/2008	Florida clade 2	GU045278.1	ACX48080.1	–		–	–
Lanarkshire/6/2008	Florida clade 2	GU045279.1	ACX48081.1	–		–	–
Leicestershire/1/2008	Florida clade 2	GU045280.1	ACX48082.1	–		–	–
Lincolnshire/1/2010	Florida clade 2	KF026381.1	AGN51041.1	–		–	–
Lothian/1/2008	Florida clade 2	GU045281.1	ACX48083.1	–		–	–
Meath/1/2007	Florida clade 2	JN222935.1	AEK98497.1	MG586816.1		–	–
Newmarket/5/2003	Florida clade 2	FJ375213.1	ACI48804.1	FJ375224.1		–	–
Northumberland/1/2008	Florida clade 2	GU045282.1	ACX48084.1	–		–	–
Perthshire/1/2009	Florida clade 2	GU045287.1	ACX48089.1	–		–	–
Perthshire/2/2009	Florida clade 2	GU045288.1	ACX48090.1	–		–	–
Richmond/1/2007	Florida clade 2	FJ195395.3	ACH95569.3	KF559336.1		–	–
Stirlingshire/1/2016	Florida clade 2	EPI957634		EPI957635		Animal Health Trust, UK	Animal Health Trust, UK
Surrey/1/2011	Florida clade 2	KF026384.1	AGN51044.1	–		–	–
Wildeshausen/1/2008	Florida clade 2	GU045273.1	ACX48075.1	–		–	–
Worcestershire/1/2008	Florida clade 2	GU045270.1	ACX48072.1	–		–	–
Xuzhou/01/2013	Florida clade 2	KF806985.1	AHA98354.1	KF806987.1		–	–
Yokohama/aq6/2012	Florida clade 2	AB761396.1	BAM66404.1	–		–	–
Yorkshire/3/2009	Florida clade 2	CY054288.1	ADM64574.1	–		–	–

a Please note that that all GISAID accession numbers begin with EPI whereas all the remaining accession numbers are from Genbank.

## 3 RESULTS

From the 25 nasopharyngeal swabs collected from horses in the quarantine area, EIV RNA was detected in 24 of them. EIV RNA was detected by rRT‐PCR targeting a portion of the gene encoding the matrix protein (M) from Influenza A viruses. The ESPLINE^TM^ Influenza A & B‐N Test Kit, a rapid detection kit for both Influenza A and B antigens, was also used to confirm the presence of the influenza A virus antigen in the nasopharyngeal swab suspension in 20 min with reactivity determined by visual colour development. The use of rRT‐PCR and rapid test methods allowed for a quick diagnosis of EIV and immediate reporting of disease. EIV was successfully isolated in chicken embryonated eggs from 5 out of 24 samples after 2 serial passages. Successful isolation was confirmed using the haemagglutination assay and by rRT‐PCR and isolates were used for further characterization.

The HA subtype was confirmed to be H3 and full‐length nucleotide sequences of HA and NA were obtained using M13 primers in Sanger sequencing. The HA sequence of A/eq/Malaysia/M201‐1/2015 (H3N8) isolate shared a nucleotide difference at position 808 as compared to A/eq/Malaysia/M201‐2/2015 (H3N8) isolate, although this nucleotide difference does not translate into any difference in amino acid sequence. The NA sequences of both isolates do not share any nucleotide difference. The nucleotide diversity among the two isolates demonstrated that there were occurrences of quasispecies during the outbreak and may suggest the presence of variant evolution during outbreaks. Two HA and one NA sequences obtained from the five isolates were deposited into GenBank (Accession numbers KT832068, KT832069 and KT867256).

**Figure 1:**
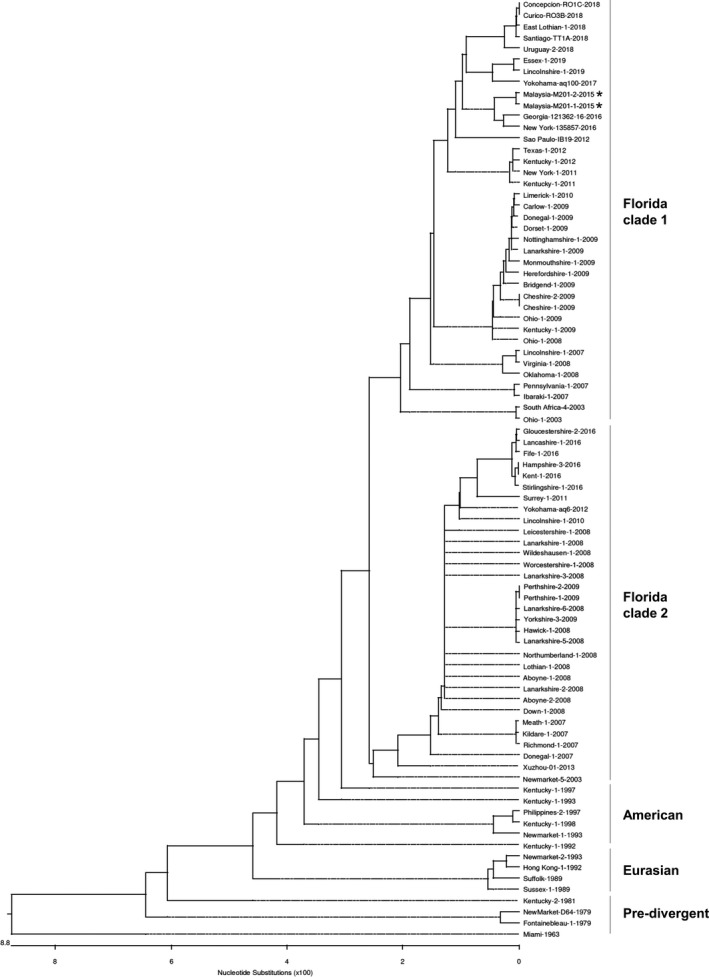
Phylogenetic analysis of HA sequences encoded by EIV subtype H3N8. The phylogenetic tree depicts five major clusters of global EIVs as indicated by the bars on the right. The Malaysia isolates (marked by an asterisk [*]) are found in the Florida clade 1 cluster. GenBank and GISAID accession numbers for the sequences are listed in Table 2

For phylogenetic analysis, representative equine H3N8 HA nucleotide sequences from the Pre‐divergent, Eurasian, American as well as Florida sub‐lineage clades 1 and 2 were retrieved from GenBank and GISAID. The phylogenetic tree demonstrated five distinct clusters corresponding to the Pre‐divergent, Eurasian lineage and American lineage as well as Florida sub‐lineage clades 1 and 2 (Figure 1). All the HA sequences from the outbreak were located within the Florida sub‐lineage clade 1 which included isolates from the USA during 2011–2012 as well as the vaccine strains (A/eq/South Africa/04/2003‐like or A/eq/Ohio/2003‐like). This suggests that a H3N8 EIV of the Florida clade 1 was responsible to be the causative agent of the influenza‐like clinical signs.

**Figure 2:**
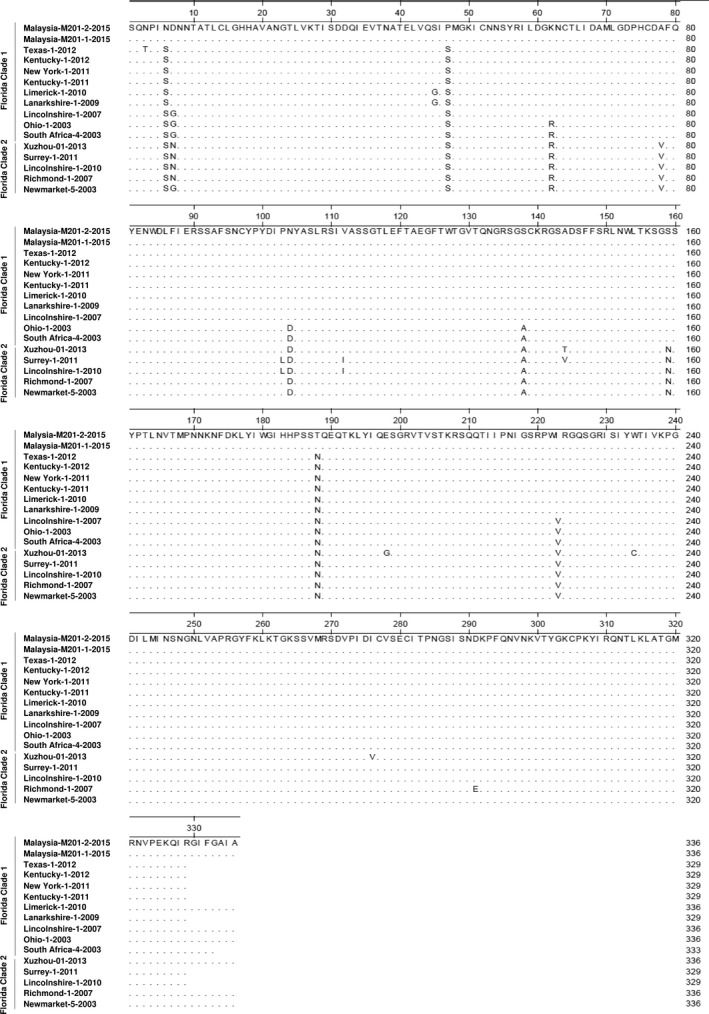
HA amino acid differences between the Malaysia isolates and representative Florida clade 1 strains (A/eq/South Africa/04/2003‐like and A/eq/Ohio/2003‐like) and clade 2 strain (A/eq/Richmond/1/2007‐like)

The derived amino acid sequences of HA for the Malaysia isolates were compared to the representative Florida clade 1 strains (A/eq/South Africa/04/2003‐like or A/eq/Ohio/2003‐like) and clade 2 strain (A/eq/Richmond/1/2007‐like). Within HA1, eight amino acid substitutions (S6N, G7D, S47P, R62K, D104N, A138S, N188T and V223I) were observed between the Malaysia isolates and the representative Florida clade 1 strains. The amino acid substitutions V78A and N159S that allow differentiation between Florida clade 1 and Florida clade 2 are also present (Figure 2).

Of the eight amino acid substitutions, the substitutions R62K, D104N, A138S and V223I have been previously described in Florida clade 1 strains (Alves Beuttemmuller et al., 2016; Legrand, Pitel, Cullinane, Fortier, & Pronost, 2014; Woodward et al., 2014), reaffirming the presence of a Florida clade 1 strain in the Malaysia outbreak. In addition, the G7D (site D), R62K (site E), D104N and A138S (site A) substitutions are located within antigenic sites which could potentially lead to reduced effectiveness of the vaccine or lead to vaccine breakdown.

**Figure 3:**
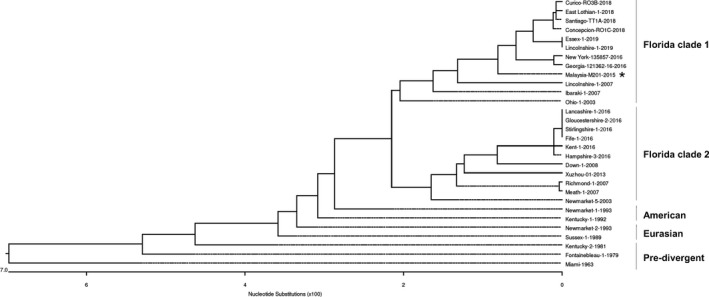
Phylogenetic analysis of NA sequences encoded by EIV subtype H3N8. The phylogenetic tree depicts five major clusters of global EIVs as indicated by the bars on the right. The Malaysia isolates (marked by an asterisk [*]) are found in the Florida clade 1 cluster. GenBank and GISAID accession numbers for the sequences are listed in Table 2

Similarly, for phylogenetic analysis of the NA gene, available equine H3N8 NA nucleotide sequences from the Pre‐divergent, Eurasian, American as well as Florida sub‐lineage clades 1 and 2 were retrieved from GenBank and GISAID. The NA sequence obtained from the outbreak clustered within the Florida sub‐lineage clade 1 together with the vaccine strains (A/eq/South Africa/04/2003‐like or A/eq/Ohio/2003‐like) (Figure 3). This reaffirms that a H3N8 EIV of the Florida clade 1 was responsible for the influenza‐like clinical signs observed in the horses.

## 4 DISCUSSION

Of the 25 clinically ill horses sampled during the Malaysia outbreak in 2015, EIV (H3N8) was detected in 24 horses. Out of which, five isolates were sequenced and two genetic variants (A/eq/Malaysia/M201‐1/2015 and A/eq/Malaysia/M201‐2/2015) were identified. The two genetic variants shared a nucleotide difference at position 808 that did not translate into any amino acid difference in the HA protein, while no sequence difference in the NA gene segment was identified between the two isolates. When the HA amino acid sequences were compared with Florida clade 1 and clade 2 strains, eight other amino acid substitutions were observed between the Malaysia isolates and Florida clade 1 strains, indicating the continued evolution of these viruses. Some of these amino acid substitutions can be found in the antigenic site which may in turn have an impact on vaccine efficacy.

Based on the current OIE recommendations, it is not necessary to include an H7N7 virus or an H3N8 virus of Eurasian lineage but they should contain both Florida clade 1 (A/eq/South Africa/04/2003‐like or A/eq/Ohio/2003‐like) and clade 2 (A/eq/Richmond/1/2007‐like). The study has identified a S6N amino acid substitution that suggests a further evolution from the G6S substitution in the Pre‐divergent, Eurasian lineage, American lineage and the Florida sub‐lineage clades 1 and 2 (Woodward, Rash, Medcalf, Bryant, & Elton, 2015). It is unknown how many substitutions can make up a genetic drift in EIV to cause a vaccine breakdown in the field but it is important to emphasize that continued surveillance is necessary to monitor the virus strains which are in circulation.

Laboratory testing plays a critical role in confirming a clinical diagnosis for EIV infection as well as in routine surveillance programmes. The rRT‐PCR probe‐based assay was used as the primary method of diagnosis during this study and subtyping/clade differentiation was complemented by sequencing methods. Through the use of specific primers in rRT‐PCR, the PCR assay allows the detection and identification of EIV from nasopharyngeal swabs in a rapid and highly sensitive manner, allowing quick clinical diagnosis to be made. Though both conventional RT‐PCR and rRT‐PCR are increasingly being used for EIV detection due to its high sensitivity and specificity, it remains important to examine the genetic sequence of emerging EIV isolates and evaluate genetic drift in the field.

In conclusion, the outbreak of respiratory disease reported in the Malaysian horses was caused by a Florida clade 1 virus. There was no indication that the virus was particularly virulent as majority of horses recovered clinically in a relatively short period of time. This raises the importance of mandatory routine surveillance systems for emerging EIV strains through advances in diagnosis and this can serve as an early warning system to facilitate implementation of proper prophylactic and control measures.

## CONFLICT OF INTEREST

The authors declare that they have no competing interests.
